# Fulminant *Brucella melitensis* Infection Presenting With Severe Cholestatic Hepatitis, Acute Kidney Injury, and Disseminated Intravascular Coagulation: A Case Report

**DOI:** 10.1155/crdi/6147749

**Published:** 2026-09-04

**Authors:** Faruk Yıldız, Esra Manici, Habibe Tülin Elmaslar Mert, Volkan İnal

**Affiliations:** ^1^ Department of Internal Medicine, Division of Intensive Care Medicine, Trakya University Faculty of Medicine, Edirne, Türkiye, trakya.edu.tr; ^2^ Department of Infectious Diseases, Trakya University Faculty of Medicine, Edirne, Türkiye, trakya.edu.tr

**Keywords:** acute kidney injury, *Brucella melitensis*, brucellosis, cholestatic hepatitis, disseminated intravascular coagulation, zoonosis

## Abstract

Brucellosis commonly manifests with fever, arthralgia, and hepatosplenomegaly, whereas severe cholestatic hepatitis is exceedingly rare. We report a 54‐year‐old goat farmer who presented with a 4‐day history of right upper quadrant tenderness, progressive jaundice, and generalized pruritus after consuming unpasteurized milk from his own herd, with which he also had daily close contact. Examination showed scleral icterus and mild lethargy without peritoneal signs. Laboratory investigation revealed profound, predominantly conjugated hyperbilirubinemia (total 25.1 mg/dL, direct 23 mg/dL) with a cholestatic enzyme pattern (alkaline phosphatase 565 U/L, gamma‐glutamyl transferase 225 U/L; R factor 0.4) and only mild transaminase elevation, together with acute kidney injury, thrombocytopenia with an elevated mean platelet volume and no platelet aggregates, hypofibrinogenemia, a markedly elevated D‐dimer, and scant schistocytes. Thrombotic microangiopathy was initially considered, but a positive direct antiglobulin test, overt coagulopathy, and absent reticulocytosis favored sepsis‐associated disseminated intravascular coagulation, confirmed by an International Society on Thrombosis and Haemostasis score of 6. Marked hyperferritinemia prompted consideration of hemophagocytic lymphohistiocytosis, which was not substantiated. Cross‐sectional abdominal imaging excluded mechanical obstruction and showed hepatosplenomegaly with subcentimeter splenic nodules and no hepatic focal lesions. *Brucella melitensis* grew from all three blood cultures, and Wright agglutination was positive at a titer of 1/160. Echocardiography excluded endocarditis, and magnetic resonance imaging excluded spondylodiscitis. Continuous venovenous hemodiafiltration was required for life‐threatening uremia, and doxycycline plus rifampin was administered for 6 weeks, with near‐complete biochemical normalization by the end of treatment. Brucellosis should be considered in patients with animal exposure who present with severe cholestatic jaundice and multiorgan involvement, since prompt combination antimicrobial therapy can achieve full recovery.

## 1. Introduction

Brucellosis is a zoonotic infection caused by Gram‐negative bacilli of the genus *Brucella*. It is endemic worldwide and constitutes a significant public health problem, particularly in communities engaged in animal husbandry, with more than 500,000 new cases estimated annually [1]. Türkiye, located in the Mediterranean basin, is among the highest‐incidence countries, where brucellosis remains endemic and is considered to be substantially underdiagnosed and underreported [2]. Transmission occurs through several routes, including ingestion of unpasteurized dairy products, direct contact of skin or mucous membranes with birth or abortion material, and inhalation of infectious aerosols in confined animal housing [3].

The clinical spectrum is broad, ranging from subclinical infection to acute febrile illness and from chronic fatigue–like presentations to localized complications such as osteoarticular, genitourinary, and neurological involvement [3]. Hepatic involvement is common but typically manifests as granulomatous hepatitis or mild transaminase elevation. By contrast, severe cholestatic hepatitis with profound conjugated hyperbilirubinemia is exceedingly rare and has been documented only in isolated reports [4]. Renal involvement is also uncommon and may present as acute tubular necrosis, interstitial nephritis, or glomerulonephritis. The concomitant occurrence of acute kidney injury (AKI) with disseminated intravascular coagulation (DIC) and thrombocytopenia represents an atypical presentation that can substantially complicate diagnosis. DIC is a severe complication of brucellosis associated with increased mortality and is typically linked to endotoxemia, cytokine release, and vascular endothelial damage [5].

This report describes an atypical, fulminant presentation of *Brucella melitensis* infection in a 54‐year‐old male goat farmer, in which the dominant and most unusual feature was severe intrahepatic cholestasis with a direct bilirubin of 23 mg/dL, accompanied by severe AKI requiring renal replacement therapy and overt DIC. Because the initial picture mimicked thrombotic thrombocytopenic purpura–hemolytic uremic syndrome (TTP–HUS) and marked hyperferritinemia additionally raised the possibility of hemophagocytic lymphohistiocytosis (HLH), the case also illustrates a practical framework for resolving these overlapping differential diagnoses at the bedside. This report was prepared in accordance with the CARE reporting guideline, and the completed CARE checklist is provided as Supporting Information 1.

## 2. Case Presentation

A 54‐year‐old male engaged in livestock farming, with no history of chronic illness or medication use, presented to the emergency department with a 4‐day history of right upper quadrant tenderness, progressive jaundice, and generalized pruritus. He kept his own herd of goats and regularly consumed unpasteurized milk from these animals. His occupation entailed daily close contact with the herd within a confined barn, and contact with abortion material was considered probable, although he had not attended any parturition. The vaccination and screening status of the herd was not documented. There was no accompanying fever, chills, or other systemic symptoms. There was no family history of hepatic, renal, or hematologic disease, and no known relevant genetic predisposition. He reported no recent travel, no alcohol or illicit drug use, and no use of hepatotoxic, over‐the‐counter, or herbal products.

On examination, prominent scleral icterus was observed. The patient was mildly lethargic, with a Glasgow Coma Scale score of 14. Vital signs were as follows: temperature 37.8°C, blood pressure 115/70 mmHg, heart rate 92 beats/min, and respiratory rate 18 breaths/min. Abdominal examination revealed localized right upper quadrant tenderness without guarding, rebound tenderness, or Murphy’s sign. Cardiac auscultation disclosed a prominent pansystolic murmur at the mitral focus, and locomotor examination identified lumbar tenderness. The respiratory examination was unremarkable.

Laboratory findings on admission are summarized in Table 1. The liver‐enzyme profile showed a cholestatic pattern, with a marked elevation of alkaline phosphatase (ALP; 565 U/L) and gamma‐glutamyl transferase (GGT; 225 U/L) and only mild transaminase elevation. The peripheral blood smear demonstrated 1%–2% schistocytes, and the clinical pathologist specifically reported the absence of platelet aggregates, thereby excluding pseudothrombocytopenia due to platelet clumping and confirming true thrombocytopenia. The mean platelet volume (MPV) was elevated at 11.9 fL. The absolute reticulocyte count was 41 × 10^9^/L, indicating an absence of reticulocytosis despite anemia. Ferritin exceeded 2000 μg/L, whereas triglycerides were 180 mg/dL.

**TABLE 1:** Laboratory findings on admission.

Parameter	Value on admission	Reference range
Total bilirubin	25.1 mg/dL	< 1.2
Direct bilirubin	23 mg/dL	< 0.3
Indirect bilirubin	2.1 mg/dL	< 0.9
Aspartate aminotransferase	57 U/L	5–34
Alanine aminotransferase	72 U/L	7–41
Alkaline phosphatase	565 U/L	40–129
Gamma‐glutamyl transferase	225 U/L	8–61
Creatinine	11.52 mg/dL	0.7–1.3
Urea	373 mg/dL	15–40
Hemoglobin	11 g/dL	13.5–17.5
Reticulocytes	1% (absolute 41 × 10^9^/L)	25–100 × 10^9^/L
Platelet count	55 × 10^9^/L	150–400
Mean platelet volume	11.9 fL	7.5–11.5
Prothrombin time	16.8 s	11–13.5
INR	1.45	0.8–1.2
Activated partial thromboplastin time	39 s	25–35
Fibrinogen	77 mg/dL	200–400
D‐dimer	11.79 mg/L FEU	< 0.5
Lactate dehydrogenase	824 U/L	135–225
Haptoglobin	1.2 mg/dL	30–200
Ferritin	> 2000 μg/L	30–400
Triglycerides	180 mg/dL	< 150
Direct antiglobulin test	Positive (IgG 2+)	Negative
Peripheral blood smear	1%–2% schistocytes; no platelet aggregates	—

*Note:* Reference ranges are those of our institutional laboratory.

Abbreviations: FEU, fibrinogen‐equivalent units; INR, international normalized ratio.

The combination of thrombocytopenia and schistocytes initially raised concern for a thrombotic microangiopathy. However, although markers of hemolysis were present (haptoglobin 1.2 mg/dL; lactate dehydrogenase 824 U/L), the anemia was only mild without reticulocytosis, the hyperbilirubinemia was predominantly conjugated, the direct antiglobulin test was positive, and coagulation was markedly deranged (a constellation inconsistent with TTP–HUS and indicative of sepsis‐associated DIC).

Cross‐sectional abdominal imaging with ultrasonography and magnetic resonance cholangiopancreatography (MRCP) demonstrated no biliary dilatation or other evidence of mechanical cholestasis. Hepatosplenomegaly was confirmed, with a liver span of 21 cm and a spleen of 14 cm. No focal hepatic lesion, abscess, or microabscess was identified. The splenic parenchyma showed a diffuse nodular appearance, the largest nodule measuring 1 cm, and subcentimeter lymphadenopathy was noted at the portal hilum and in the para‐aorto‐caval region.

**FIGURE 1:**
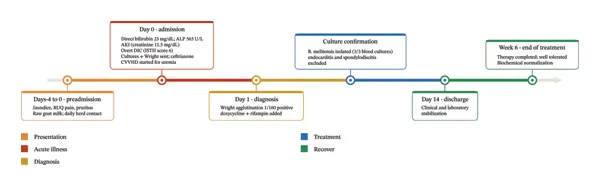
Timeline of the clinical course. AKI, acute kidney injury; CVVHD, continuous venovenous hemodiafiltration; DIC, disseminated intravascular coagulation; ISTH, International Society on Thrombosis and Haemostasis.

To manage life‐threatening uremia, the patient underwent continuous venovenous hemodiafiltration (CVVHD) as renal replacement therapy, delivered with a sepsis adsorption filter in predilution mode without systemic anticoagulation, for a total of 48 h over two consecutive days. On the day of admission, three sets of blood cultures and a Wright agglutination test were obtained, and empirical ceftriaxone (2 g/day intravenously) was started. Empirical therapy was considered justified despite the absence of frank fever because the patient was subfebrile with severe multiorgan dysfunction, and leptospirosis (which characteristically presents with jaundice and AKI and is endemic in the region) was a leading alternative diagnosis. On Day 1, the Wright agglutination test returned positive at a titer of 1/160, and oral doxycycline (100 mg twice daily) and rifampin (600 mg/day) were added. All three blood cultures subsequently grew *B. melitensis*, confirming the diagnosis. Because isolation from three blood cultures indicated significant bacteremia, infective endocarditis was excluded by transthoracic echocardiography in accordance with the modified Duke criteria [6]; no vegetation, valvular dysfunction, or other evidence of endocarditis was found. Serological testing for leptospirosis and vasculitides was negative, and lumbar pain prompted contrast‐enhanced magnetic resonance imaging, which excluded spondylodiscitis. Progressive improvement in bilirubin and creatinine followed (Table 2), and the overall clinical course is summarized in Figure 1.

**TABLE 2:** Changes in laboratory parameters over time.

Parameter	Day 0 (admission)	Day 1	Day 3	Day 14 (discharge)	Week 6 (end of treatment)
Total bilirubin (mg/dL)	25.1	22.1	19.3	1.3	0.9
Direct bilirubin (mg/dL)	23	22	18.9	1.2	0.8
Alkaline phosphatase (U/L)	565	515	539	142	140
Gamma‐glutamyl transferase (U/L)	225	225	176	59	54
Creatinine (mg/dL)	11.52	7.3	2.52	0.9	1.07
Urea (mg/dL)	373	242	93	46	53
Platelets (× 10^9^/L)	55	43	30	436	413
INR	1.45	1.36	1.39	1.0	1.15

Abbreviation: INR, international normalized ratio.

### 2.1. Follow‐Up and Outcomes

The patient was monitored as an inpatient for 2 weeks and discharged after clinical and laboratory stabilization. He completed the remaining course of doxycycline plus rifampin on an outpatient basis, for a total treatment duration of 6 weeks. The regimen was well tolerated: He reported no gastrointestinal symptoms, no photosensitivity or other cutaneous reactions, and full adherence to the prescribed course. Cholestatic parameters were still improving at discharge (ALP 142 U/L, GGT 59 U/L, and total bilirubin 1.3 mg/dL). Laboratory parameters repeated at the end of treatment showed near‐complete normalization, with total bilirubin 0.9 mg/dL, direct bilirubin 0.8 mg/dL, creatinine 1.07 mg/dL, urea 53 mg/dL, INR 1.15, platelets 413 × 10^9^/L, GGT 54 U/L, and ALP 140 U/L, the last remaining marginally above the upper reference limit (Table 2). Abdominal imaging was not repeated after discharge, so radiological resolution of the hepatosplenomegaly could not be documented. No relapse or other complication was observed during follow‐up.

## 3. Discussion

Brucellosis typically presents with subacute fever, arthralgia, and hepatosplenomegaly. The distinguishing feature of the present case was the magnitude of cholestasis (a direct bilirubin of 23 mg/dL) occurring together with severe AKI and overt DIC [1].

Hepatic involvement is common in brucellosis but usually takes the form of granulomatous hepatitis or mild transaminitis; cases with direct hyperbilirubinemia above 15 mg/dL are seldom reported [4, 7]. In this patient, ALP was elevated to approximately 4.4 times and GGT to approximately 3.7 times the upper reference limit, with only mild transaminase elevation. The corresponding R factor of 0.4 (well below the threshold of 2) confirmed a cholestatic rather than hepatocellular pattern of injury. Serial measurements defined the time course of the cholestasis (Table 2). ALP and GGT plateaued over the first three days (ALP 565, 515, and 539 U/L; GGT 225, 225, and 176 U/L) and declined only thereafter, reaching 142 and 59 U/L by discharge on Day 14 and 140 and 54 U/L at the end of treatment, with ALP remaining marginally above the upper reference limit at both time points. Renal recovery was considerably faster, creatinine falling from 11.52 to 2.52 mg/dL by Day 3. This dissociation between rapid restoration of renal function and delayed resolution of cholestasis is characteristic of infection‐associated intrahepatic cholestasis, in which recovery of canalicular transport lags behind control of the underlying infection, and it accounts for the persistently abnormal hepatic parameters at the time of discharge. After mechanical obstruction had been excluded by ultrasonography and MRCP, and in the absence of focal hepatic lesions or microabscesses, the findings were interpreted as intrahepatic cholestasis. Plausible mechanisms include direct injury to the canalicular membrane, granulomatous compression of intrahepatic bile ductules, and cytokine‐mediated downregulation of hepatobiliary transport proteins, the last being the accepted mechanism of sepsis‐associated cholestasis [7]. Serum bile acid measurement, which would have further characterized the cholestasis, is not available at our institution, and a next‐generation sequencing panel for inherited cholestatic syndromes was not performed. Nevertheless, the acute onset at the age of 54 without prior episodes, the concurrent systemic infection, and the complete biochemical normalization after antimicrobial therapy render an inherited cholestatic disorder highly improbable. Drug‐induced liver injury was likewise considered unlikely, since the patient had taken no prescription, over‐the‐counter, or herbal products and did not consume alcohol; the rapid resolution of cholestasis in parallel with antimicrobial therapy further supports an infectious rather than a toxic etiology.

The combination of splenomegaly, cytopenia, hypofibrinogenemia, and a ferritin concentration above 2000 μg/L raised the possibility of secondary HLH [8], which has been described in association with brucellosis [9]. Applying the HLH‐2004 criteria, the patient fulfilled three of eight: splenomegaly, hypofibrinogenemia (0.77 g/L), and ferritin ≥ 500 μg/L. He did not meet the fever criterion (37.8°C), and his triglycerides of 180 mg/dL were below the diagnostic threshold of 265 mg/dL, and cytopenia affected only one lineage, since hemoglobin was 11 g/dL rather than below 9 g/dL. Soluble interleukin‐2 receptor, natural killer cell activity, and bone marrow examination were not obtained, so HLH cannot be formally excluded. However, several considerations argue against clinically significant HLH: Hyperferritinemia is a nonspecific acute‐phase response in severe sepsis, the hypofibrinogenemia was adequately explained by consumptive coagulopathy, and, most importantly, the patient recovered completely on antimicrobial therapy alone, without corticosteroids, immunoglobulin, or etoposide. This distinction is clinically consequential, since a mistaken diagnosis of HLH would have exposed the patient to unnecessary immunosuppression during active bacteremia.

Renal involvement in brucellosis is uncommon and may manifest as prerenal azotemia, tubulointerstitial nephritis, or glomerulonephritis [10]. In this patient, a creatinine exceeding 10 mg/dL with urea above 350 mg/dL indicated severe AKI with life‐threatening uremia, for which CVVHD was initiated as renal replacement therapy. A sepsis adsorption filter was incorporated on the theoretical rationale that Gram‐negative sepsis is mediated in part by endotoxin and proinflammatory cytokines and that extracorporeal blood‐purification techniques may modulate this response. However, several considerations temper any causal interpretation: The patient was hemodynamically stable and not in septic shock (blood pressure 115/70 mmHg, no vasopressor requirement); endotoxin and cytokine levels were not measured; and clinical improvement coincided with the initiation of effective combination antibiotic therapy, which remains the established curative treatment for brucellosis. The independent contribution of the adsorption filter therefore cannot be determined from a single case and remains speculative. Evidence for extracorporeal endotoxin removal (for example, polymyxin B hemoperfusion) derives from selected subgroups of patients with Gram‐negative septic shock and has produced inconsistent results in randomized controlled trials [11]; no data specific to *Brucella* infection exist, and extrapolation to the present setting should be regarded as hypothesis‐generating only.

Thrombocytopenia and schistocytes initially raised suspicion for TTP–HUS, but several features supported DIC. Although hemolysis was present, the direct antiglobulin test was positive (IgG 2+), indicating an immune‐mediated hemolytic component rather than the Coombs‐negative microangiopathic hemolysis that defines TTP–HUS. Coagulation was overtly deranged, whereas TTP–HUS characteristically preserves coagulation; the anemia was only moderate, and schistocytes were scant (1%–2%). Two additional hematological observations supported peripheral consumption rather than marrow failure. First, the absence of platelet aggregates on the smear confirmed that the thrombocytopenia was genuine rather than artefactual. Second, the MPV was elevated at 11.9 fL; an increased MPV reflects the release of larger, younger platelets in response to peripheral destruction or consumption, and has been proposed as a discriminator between destructive and hypoproductive thrombocytopenia, with the majority of patients in overdestruction cohorts exhibiting values at or above 8.8 fL [12]. Applying the International Society on Thrombosis and Haemostasis (ISTH) overt‐DIC algorithm to the presenting values (platelet count 55 × 10^9^/L [1 point], markedly elevated D‐dimer indicating a strong increase in a fibrin‐related marker [3 points], prothrombin time prolonged approximately 3.3 s beyond the upper reference limit [1 point], and fibrinogen 0.77 g/L [1 point]) yielded a total of 6 points in the setting of a recognized DIC‐associated condition, exceeding the threshold of ≥ 5 that defines overt DIC. ADAMTS13 activity was not measured, which is acknowledged as a limitation; nonetheless, the combination of an overt‐DIC ISTH score, Coombs‐positive hemolysis, and *Brucella* bacteremia established DIC rather than TTP–HUS as the underlying process.

The diffuse subcentimeter nodularity of the splenic parenchyma, together with subcentimeter abdominal lymphadenopathy, is consistent with the reticuloendothelial tropism of *Brucella*, which characteristically produces granulomatous foci in the spleen, liver, and lymph nodes. The absence of hepatic focal lesions or microabscesses argues against a suppurative hepatic process and is consistent with a diffuse cholestatic rather than space‐occupying pattern of liver involvement. Because abdominal imaging was not repeated after discharge, resolution of the organomegaly could not be documented; radiological regression typically lags behind clinical and biochemical recovery, and follow‐up imaging would have strengthened the report.

The patient kept goats, which is epidemiologically coherent, since *B. melitensis* is primarily adapted to sheep and goats, whereas *B. abortus* is chiefly a pathogen of cattle, notwithstanding recognized cross‐infection. Although consumption of unpasteurized goat milk is the most conspicuous exposure, it should not be assumed to be the only or even the principal route. *Brucella* species are readily aerosolized and have a very low infectious dose, estimated at 10–100 organisms, so inhalation of contaminated aerosols within a confined barn and percutaneous or mucosal contact with birth or abortion material are equally plausible routes in a farmer with daily herd contact [3, 13]. In this patient, the precise route could not be established, and the relative contribution of ingestion, inhalation, and direct contact remains undetermined. From a public health perspective, control of human brucellosis depends primarily on control of animal infection through vaccination of small ruminants with the live attenuated Rev‐1 strain, serological screening with test‐and‐slaughter policies, and pasteurization of milk [2]. The vaccination and screening status of this patient’s herd was not documented, which is itself instructive, since unscreened smallholder flocks represent a persistent reservoir in endemic regions.

The treatment response was prompt with doxycycline plus rifampin, and the 6‐week duration aligns with World Health Organization recommendations [14]. Although doxycycline plus rifampin is a widely used oral regimen, meta‐analytic data suggest that a doxycycline–aminoglycoside combination may be associated with lower relapse and treatment‐failure rates [15]. Early empirical ceftriaxone provided coverage against Gram‐negative organisms, including leptospirosis, during the initial diagnostic phase. Doxycycline at 200 mg daily for 6 weeks carries a recognized risk of gastrointestinal intolerance and photosensitivity, neither of which occurred in this patient. Because relapse occurs in a minority of patients and typically within the first year, clinical and serological follow‐up at approximately 6 and 12 months after completion of therapy is advisable; a rising or persistently elevated agglutination titer, particularly in conjunction with recurrent symptoms, should prompt reevaluation for relapse [14, 15].

Multisystem brucellosis with DIC has been reported previously, most recently by Zhang et al., who described DIC with liver injury [5]; a comparison with that and other relevant reports is presented in Table 3. The present case is distinguished less by the occurrence of DIC than by the severity of the cholestatic hepatic injury, with a direct bilirubin of 23 mg/dL, and by the need for renal replacement therapy (a combination not documented in the cases identified). In the published reports, treatment consisted of prolonged combination antibiotic therapy, most commonly doxycycline plus rifampin, tailored to severity, with transfusion support in severe DIC, and dialysis was rarely required.

**TABLE 3:** Comparison with similar reported cases of *B. melitensis* infection in the literature.

Case (year, reference)	Complications	Blood culture	Treatment	Outcome
Present case	Severe cholestatic hepatitis (direct bilirubin 23 mg/dL) + AKI requiring CVVHD + overt DIC (ISTH 6)	3/3 (+)	Empirical ceftriaxone, then doxycycline + rifampin (6 weeks) + CVVHD	Complete clinical recovery; biochemical normalization at 6 weeks
Zhang et al. (2025) [5]	DIC + liver injury	(+)	Ceftriaxone, then doxycycline + meropenem	Recovery; DIC and liver injury resolved
García Casallas et al. (2018) [4]	Acute liver failure	(−)	Doxycycline + rifampin (6 weeks) + supportive care	Liver function recovered
Ceylan et al. (2009) [10]	Renal involvement (15 patients; 14 with renal failure)	Variable	Antibiotics ± hemodialysis	Renal recovery in 11/15

*Note:* CVVHD, continuous venovenous hemodiafiltration.

Abbreviations: AKI, acute kidney injury; DIC, disseminated intravascular coagulation; ISTH, International Society on Thrombosis and Haemostasis.

### 3.1. Limitations

This is a single case report, which limits generalizability. Serum bile acids could not be measured, as the assay is unavailable at our institution, and a genetic panel for inherited cholestasis was not performed. Liver biopsy was not undertaken, so the histological pattern of hepatic involvement could not be confirmed. Soluble interleukin‐2 receptor, natural killer cell activity, and bone marrow examination were not obtained, so HLH cannot be formally excluded, and ADAMTS13 activity was not measured. Endotoxin and cytokine concentrations were not measured, so the contribution of the sepsis adsorption filter cannot be established. Abdominal imaging was not repeated after discharge. Finally, the exact route of transmission and the vaccination status of the herd could not be determined.

## 4. Conclusion

This case documents an unusually severe cholestatic presentation of *B. melitensis* infection, with a direct bilirubin of 23 mg/dL, accompanied by profound renal failure and overt DIC. Brucellosis should be prioritized in the differential diagnosis of patients with animal exposure who present with severe cholestatic jaundice and multiorgan involvement, particularly in endemic regions. A structured bedside approach (the ISTH score, the direct antiglobulin test, and platelet indices to separate DIC from TTP–HUS, and the HLH‐2004 criteria to weigh hyperferritinemia) allows these overlapping syndromes to be resolved without recourse to empirical immunosuppression. Early blood cultures, serological testing, and prompt targeted combination antibiotic therapy are central to recovery, and renal replacement therapy with CVVHD is indicated for severe brucellosis‐associated AKI with life‐threatening uremia, whereas the additional role of extracorporeal blood‐purification techniques remains unproven. In endemic regions, animal vaccination and screening, promotion of pasteurized‐milk consumption, and public education (including awareness of nonoral routes of transmission) remain essential to reduce disease burden.

## Author Contributions

F.Y. was responsible for the intensive care management of the patient, data collection, and drafting of the manuscript. E.M. and H.T.E.M. were responsible for the diagnostic evaluation and antimicrobial management of the infection and critically revised the manuscript. V.İ. supervised the clinical management of the patient and critically revised the manuscript for important intellectual content.

## Funding

The authors received no specific funding for this work.

## Disclosure

All authors have read and approved the final version of the manuscript and agree to be accountable for all aspects of the work.

## Ethics Statement

In accordance with the policy of our institution, ethics committee approval is not required for single‐patient case reports. The report was prepared in accordance with the principles of the Declaration of Helsinki.

## Consent

Written informed consent for publication of this case report and any accompanying data was obtained from the patient. A copy of the signed consent form is available for review by the editor on request. The report has been anonymized, and identifying details have been removed in accordance with ICMJE recommendations.

## Conflicts of Interest

The authors declare no conflicts of interest.

## Supporting Information

Additional supporting information can be found online in the Supporting Information section.

## Supporting information


**Supporting Information** Supporting Information 1: Completed CARE checklist for this case report.

## Data Availability

The data that support the findings of this study are available on request from the corresponding author. The data are not publicly available due to privacy or ethical restrictions.
