# Supplementary material for: Fulminant Brucella melitensis Infection Presenting With Severe Cholestatic Hepatitis, Acute Kidney Injury, and Disseminated Intravascular Coagulation: A Case Report
Source: Case Rep Infect Dis. 2026 Sep 4;2026:6147749. doi: 10.1155/crdi/6147749 (PMC13544422; doi:10.1155/crdi/6147749)
Supplement: Supplementary file 1 [file CRDI-2026-6147749-s001.docx]

**CARE Checklist (2013) — Information to include when writing a case report**

**Manuscript (revised):** “Fulminant Brucella melitensis Infection Presenting with Severe Cholestatic Hepatitis, Acute Kidney Injury, and Disseminated Intravascular Coagulation: A Case Report.”

| **Topic** | **Item** | **Checklist item description** | **Where addressed in the revised manuscript** |
| --- | --- | --- | --- |
| **Title** | 1 | Includes the words “case report” and the area of focus. | **✓** Title page |
| **Key Words** | 2 | 2–5 key words capturing the key topics/diagnoses. | **✓** Keywords, after Abstract |
| **Abstract** | 3a | Introduction — what is unique and what it adds. | **✓** Abstract, opening sentences |
|  | 3b | Main symptoms and/or important clinical findings. | **✓** Abstract |
|  | 3c | Main diagnoses, therapeutic interventions, and outcomes. | **✓** Abstract |
|  | 3d | Conclusion / main take-away lesson. | **✓** Abstract, final sentence |
| **Introduction** | 4 | One or two paragraphs on why the case is unique, with references. | **✓** Section 1 |
| **Patient Information** | 5a | De-identified demographic and patient-specific information. | **✓** Section 2, paragraph 1 |
|  | 5b | Primary concerns and symptoms of the patient. | **✓** Section 2, paragraph 1 |
|  | 5c | Medical, family, and psychosocial history, including relevant genetic information. | **✓** Section 2, paragraph 1 (occupational and dietary exposure; no family history; no genetic predisposition; no travel, alcohol, or herbal use) |
|  | 5d | Relevant past interventions and their outcomes. | **✓** Section 2, paragraph 1 (no prior medication use) |
| **Clinical Findings** | 6 | Relevant physical examination and other clinical findings. | **✓** Section 2, paragraph 2 |
| **Timeline** | 7 | Key events organized as a timeline (figure or table). | **✓** Figure 1; complemented by Table 2 |
| **Diagnostic Assessment** | 8a | Diagnostic methods (examination, laboratory, imaging). | **✓** Section 2 and Table 1 |
|  | 8b | Diagnostic challenges. | **✓** Section 3 (differentiation from TTP-HUS and HLH) |
|  | 8c | Diagnostic reasoning, including other diagnoses considered. | **✓** Section 3 (cholestasis, HLH-2004 criteria, ISTH overt-DIC score) |
|  | 8d | Prognostic characteristics where applicable. | **✓** Section 3 and Table 3 |
| **Therapeutic Intervention** | 9a | Types of intervention. | **✓** Section 2 (antimicrobials; CVVHD) |
|  | 9b | Administration (dose, strength, duration). | **✓** Section 2 |
|  | 9c | Changes in intervention, with explanation. | **✓** Section 2 (ceftriaxone → doxycycline + rifampin on day 1) |
| **Follow-up and Outcomes** | 10a | Clinician- and patient-assessed outcomes. | **✓** Section 2.1 and Table 2 |
|  | 10b | Important follow-up diagnostic and other test results. | **✓** Section 2.1 and Table 2 (serial values to the end of treatment) |
|  | 10c | Intervention adherence and tolerability. | **✓** Section 2.1 (no gastrointestinal symptoms or photosensitivity; full adherence) |
|  | 10d | Adverse and unanticipated events. | **✓** Section 2.1 (no relapse or complication) |
| **Discussion** | 11a | Strengths and limitations in management. | **✓** Section 3.1 (Limitations) |
|  | 11b | Discussion of the relevant medical literature. | **✓** Section 3 and Table 3 |
|  | 11c | Rationale for the conclusions. | **✓** Sections 3 and 4 |
|  | 11d | Primary take-away lessons. | **✓** Section 4 |
| **Patient Perspective** | 12 | The patient shares their perspective on the treatment received. | **✓*** Omitted at the reviewer's explicit request; a Follow-up and Outcomes subsection (Section 2.1) reporting tolerability, adherence, and post-treatment results was substituted. |
| **Informed Consent** | 13 | Did the patient give written informed consent? | **✓** Consent statement |

** Item 12 was omitted in the revised version at the explicit request of Reviewer 2, who considered the Patient Perspective section insufficiently scientific and recommended replacing it with patient follow-up data. A Follow-up and Outcomes subsection was added accordingly.*

*Reference: Gagnier JJ, Kienle G, Altman DG, Moher D, Sox H, Riley D; the CARE Group. The CARE guidelines: consensus-based clinical case reporting guideline development. 2013.*
